# The Circulatory Effects of Increased Hydrostatic Pressure Due to Immersion and Submersion

**DOI:** 10.3389/fphys.2021.699493

**Published:** 2021-07-19

**Authors:** Robert P. Weenink, Thijs T. Wingelaar

**Affiliations:** ^1^Diving Medical Center, Royal Netherlands Navy, Den Helder, Netherlands; ^2^Department of Anesthesiology, Amsterdam University Medical Centers, Location AMC, Amsterdam, Netherlands

**Keywords:** hydrostatic pressure, immersion, blood circulation, hyperbaric oxygenation, swimming, diving, immersion pulmonary edema, rescue collapse

## Abstract

Increased hydrostatic pressure as experienced during immersion and submersion has effects on the circulation. The main effect is counteracting of gravity by buoyancy, which results in reduced extravasation of fluid. Immersion in a cold liquid leads to peripheral vasoconstriction, which centralizes the circulation. Additionally, a pressure difference usually exists between the lungs and the rest of the body, promoting pulmonary edema. However, hydrostatic pressure does not exert an external compressing force that counteracts extravasation, since the increased pressure is transmitted equally throughout all tissues immersed at the same level. Moreover, the vertical gradient of hydrostatic pressure down an immersed body part does not act as a resistance to blood flow. The occurrence of cardiovascular collapse when an immersed person is rescued from the water is not explained by removal of hydrostatic squeeze, but by sudden reinstitution of the effect of gravity in a cold and vasoplegic subject.

## Introduction

Humans are generally subjected to a rather constant environmental pressure, but may experience changes in ambient pressure during activities such as flying and diving. One of the most common activities that involve increased environmental pressure is immersion (when the body is partially surrounded by liquid) as occurs during bathing or swimming, or submersion (when the body is fully surrounded by liquid), when snorkeling, diving, or swimming under water. When reviewing the literature on the circulatory effects of immersion and submersion one frequently encounters misconceptions, which seem to result from an incorrect application of physical principles. A regularly encountered misconception concerns the phenomenon of cardiovascular collapse when an immersed or submerged person is removed from the water (Tipton and Ducharme, 2014). This is frequently attributed to the removal of “hydrostatic squeeze,” i.e., the supposed compressing effect of hydrostatic pressure on the immersed tissue (Pendergast et al., 2015; Bierens et al., 2016). At first glance, this explanation, which suggests that hydrostatic pressure may act similarly to an elastic stocking around the leg (Tipton et al., 2017), may seem convincing. However, it does not comply with the physics involved in hydrostatic pressure. In this perspective, we will provide an overview of the effects of immersion and submersion on the circulation, address frequently encountered misconceptions, and thereby hope to encourage the correct application of physical principles and use of appropriate terminology.

## Physiology of Increased Hydrostatic Pressure

Firstly, it should be appreciated that Pascal’s law dictates that hydrostatic pressure acting on an immersed body part is transmitted equally through all tissues. In other words, the pressure at a certain depth of immersion will be equal in all tissues immersed at that level. This means that there is no pressure gradient between – for instance – the blood vessels and the surrounding tissues. Therefore, no force is squeezing the vessels, pushing interstitial fluid into the vessels, or counteracting extravasation. This is best demonstrated by regarding the effects of increasing pressure in a hyperbaric chamber. A person subjected to increased ambient pressure in a dry chamber, the pressure is equal all around and inside the body, i.e., the increased atmospheric pressure in the chamber is transmitted throughout the body. Hence, there is no pressure gradient to cause a circulatory effect. This is in line with the experience of anyone working in hyperbaric medicine: in the range of pressures encountered in the hyperbaric chamber – usually up to three atmospheres (304 kPa), equivalent to immersion in water to a depth of 20 m – the pressure *per se* has no effect (Welslau, 2006). For sake of completeness, it should be noted that other components of hyperbaric medicine can have circulatory effects, such as vasoconstriction caused by increased partial oxygen tensions (Mathieu et al., 2006), but this is not the topic of the current paper.

So far, we can conclude that the amount of pressure increase is equal in all tissues at the same level of immersion. Increased hydrostatic pressure is not the same as an elastic stocking. What then are the causes of circulatory effects due to increased hydrostatic pressure during immersion or submersion? In order to understand these, we must consider the differences between compression in a dry hyperbaric chamber and immersion or submersion in a liquid:

1. Counteracting of gravity by buoyancy.
2. Vertical pressure gradient of hydrostatic pressure.
3. Pressure gradient between the lungs and the rest of the body.
4. Effects due to temperature.
5. Miscellaneous other effects.

### Buoyancy

Archimedes’ law states that an object submerged in a liquid experiences an upward force equal to the weight of the displaced liquid. This is caused by the upward force exerted on the object by the liquid below the object being greater than the downward force exerted by the liquid above the object. As the density of the human body is quite comparable to the density of water, this upward force is almost equal to the weight of the body, and therefore humans experience “weightlessness” during submersion. In a standing, non-immersed person there is a considerable intravascular pressure gradient down the body due to gravity. If we assume a mean blood pressure of 74 mmHg (100 cm H_2_O, 9.81 kPa) in the aortic root, the mean intravascular pressure 1 m lower in the legs will be 200 cm H_2_O (19.6 kPa), promoting extravasation of fluid. During immersion, the buoyancy caused by the upward force of the liquid on the body counteracts gravity and thereby reduces the gradient for extravasation. Decreased extravasation means more fluid is retained in the circulation, and this accounts for the approximately 500–700 ml increase in circulating volume as measured during submersion (Weston et al., 1987). The increased urine output that follows will tend to normalize circulating volume over time.

### Pressure Gradient of Hydrostatic Pressure

In a hyperbaric chamber, the pressure increase is equal all around the body. During immersion and submersion, this is not the case. Non-immersed parts of the body experience the atmospheric pressure of the air above the water, while immersed parts experience higher pressures, depending on the level of immersion. If the immersion is in water, the pressure gradient will be 100 cm H_2_O (9.81 kPa) for each meter of immersion. It is exactly this pressure gradient that causes buoyancy as explained above. What now is the effect of this vertical pressure gradient, reiterating that at every given level of immersion, the pressure inside the tissues at that level is equal?

One might be inclined to think of this increase in pressure in the blood vessels and tissues as acting as a resistance to blood flow. During submersion in a vertical position, on its way from the heart to the legs the blood encounters increasingly greater pressures, resulting in incremental decreases of flow. This would then lead to preferential perfusion of the least immersed parts of the body. However, this is not the case. The reason for this is that the circulation is a siphon, i.e., a closed loop of flowing liquid without any air. In a siphon, flow is determined by the difference between the inlet and the outlet pressure and the resistance of the system; intermediary pressure has no effect (Munis and Lozada, 2000). Consider a garden hose through which water flows at a specific rate. If a portion of the hose is now lowered (or elevated) while inlet and outlet remain at the same level, the flow rate will not change. If the pressure inside the hose would be measured, it would be greater in the lower portion of the hose, but this increase in intermediate pressure does not affect flow rate.

It should be borne in mind that lowering part of the hose is not the same as squeezing the hose. Squeezing the hose is equivalent to external compression on the dependent body parts. As explained above, this is not what happens during immersion. Also, it is important to realize that the gradient of hydrostatic pressure due to immersion is different from the gradient of intravascular pressure in a standing non-immersed person. As mentioned above, in a standing non-immersed person, intravascular pressure increases on the way down from the heart, due to the effect of gravity on the blood. This has no effect on flow (again, because the circulation is a siphon) but since in this case, a pressure difference between the blood in the vessel and extravascular tissues does exist, it promotes extravasation of fluid.

### Pressure Gradient Between the Lung and the Rest of the Body

**Figure 1:**
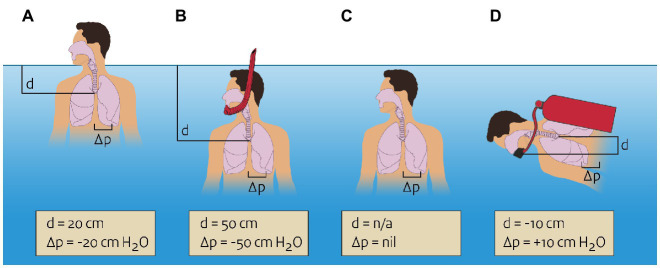
Pressures in and around the lung during various types of immersion or submersion. Negative pressure differential means the pressure in the lung is lower than the pressure in the surrounding tissue. **(A)** Head-out-water-immersion, pressure differential of −20 cm H_2_O. **(B)** snorkeling, pressure differential of −50 cm H_2_O. **(C)** Swimming under water (breath holding), no pressure differential. **(D)** Diving with mouthpiece 10 cm below the lung, pressure differential of +10 cm H_2_O.

In most situations of submersion and immersion, there will be a pressure difference between the air in the lung and the rest of the body. For instance, during immersion with the head above the water (head-out-water-immersion) and snorkeling, air pressure in the lung is equal to the atmospheric pressure of the air breathed, and the pressure in the tissue surrounding the lung depends on the level of immersion (Figure 1). With head-out-water-immersion, the lung will be some 20 cm below the water, resulting in a pressure differential of 20 cm H_2_O (2.0 kPa). The pressure gradient between the air in the lungs and the surrounding tissues promotes extravasation of fluid from the pulmonary vasculature. This is one of the supposed mechanisms in immersion pulmonary edema (Koehle et al., 2005; Wilmshurst, 2019) and is also a reason why snorkels cannot be longer than a few decimeters, or the large pressure difference would lead to pulmonary edema.

Of note, a pressure difference between the lungs and the surrounding tissues is absent when a person is fully submerged and not breathing, such as when swimming below the surface. In this case, pressure in the lung equalizes with surrounding pressure, again following Pascal’s law. During diving a pressure difference between lung and surrounding tissues may exist, depending on the pressure at which the breathing gases are delivered to the diver. Usually, some amount of resistance has to be overcome in order to draw breathing gas into the lung and expel it out of the lungs. If this resistance is too high, excessive negative airway pressure will exist during inspiration, again promoting pulmonary extravasation of fluid. Through this and other mechanisms, immersion pulmonary edema can also occur during diving (Coulange et al., 2010).

The special case of breath-hold diving must be briefly mentioned here. This is basically submersion while holding one’s breath, and therefore no pressure difference between lungs and surroundings exists. However, the volume of the air in the lungs will decrease as the diver goes deeper, per Boyle’s law. At a certain point, lung volume will reach the residual volume so the lung cannot collapse any further. Further descent causes extravasation of fluid from the pulmonary vasculature, which is a cause of pulmonary edema in extreme breath-hold diving (Lindholm and Lundgren, 2009).

### Effects Due to Temperature

All of the abovementioned changes in hydrostatic pressure have been assumed to occur in a thermoneutral environment (water of approximately 35°C). However, in most cases of immersion or submersion, this is not a realistic assumption. When immersion or submersion occurs in cold water, stimulation of autonomic nerve fibers will result in peripheral vasoconstriction in order to prevent heat loss. This will – together with the effect of buoyancy as explained above – increase centralization of circulating volume. If, on the other hand, peripheral vasodilation occurs, such as during bathing in hot water, this may counteract the centralization of circulation volume as seen in thermoneutral or cold water.

### Additional Factors to Consider

For the sake of completeness, we will briefly mention two circulatory effects that may occur during immersion or submersion, although they are not due to hydrostatic pressure and therefore not the aim of this paper. The first is the mammalian diving reflex, which consists of parasympathetic stimulation leading to bradycardia, apnea, and vasoconstriction upon facial contact with liquid (Bosco et al., 2018). Mean arterial pressure is usually increased due to the vasoconstriction, despite bradycardia. There is an inverse relationship with water temperature: the effect is stronger in cold water. The effects are transient, dissolving after approximately 5–10 min (Lundell et al., 2020).

Secondly, when a tightly fitting suit is worn, the elastic compression may exert external compression on the tissue. In this case, an active compression counteracting the extravasation of fluid is present. The amount of pressure caused by swimming or diving suits has, to our knowledge, not been scientifically determined. We estimate this could be compared to the pressure of a mild compression stocking [20–25 cm H_2_O (2.0–2.5 kPa)].

## Discussion

In summary, a few conclusions can be drawn. Firstly, immersion and submersion affect the circulation. This is caused by buoyancy, which abolishes the effect of gravity and therefore reduces extravasation that normally occurs in dependent parts of the body. This results in increased circulating volume. Peripheral vasoconstriction decreases peripheral perfusion and leads to a more centralized circulation. Additionally, when the pressure in the lungs is lower than the pressure in the surrounding tissues, there is a gradient for extravasation from the lung which may cause pulmonary edema. However, the increased hydrostatic pressure does not act as an external compressing force on immersed body parts. Even when there is a vertical pressure gradient of pressure down the body, these increased intermediary pressures encountered by the blood when flowing from the heart to the dependent parts of the body do not act to reduce blood flow.

As for the phenomenon of rescue collapse, this is not explained by the removal of a squeezing effect of hydrostatic pressure on the body. Instead, it can be explained by the sudden return of the effect of gravity. After prolonged immersion, the subject is cold and vasoplegic. After an initial increase, the circulating volume has been normalized by increased diuresis. It is not hard to imagine the profound effect that sudden reinstitution of the effect of gravity may have on such a person. Additionally, removal of a tightly fitting suit may remove its compressing effect. These effects more than suffice to explain rescue collapse and no supposed removal of hydrostatic squeeze are needed.

## Data Availability Statement

The original contributions presented in the study are included in the article/supplementary material, further inquiries can be directed to the corresponding author.

## Author Contributions

Both authors contributed to the conception of the manuscript as well as writing of the initial and subsequent versions.

### Conflict of Interest

The authors declare that the research was conducted in the absence of any commercial or financial relationships that could be construed as a potential conflict of interest.
